# Opa1 Deficiency Promotes Development of Retinal Vascular Lesions in Diabetic Retinopathy

**DOI:** 10.3390/ijms22115928

**Published:** 2021-05-31

**Authors:** Dongjoon Kim, Marcela Votruba, Sayon Roy

**Affiliations:** 1 Department of Medicine, Boston University School of Medicine, Boston, MA 02118, USA; djkim@bu.edu; 2 Department of Ophthalmology, Boston University School of Medicine, Boston, MA 02118, USA; 3 School of Optometry and Vision Sciences, Cardiff University, Cardiff CF24 4HQ, UK; votrubam@cardiff.ac.uk; 4 Cardiff Eye Unit, University Hospital of Wales, Cardiff CF14 4XW, UK

**Keywords:** Opa1, apoptosis, mitochondrial dysfunction, diabetic retinopathy

## Abstract

This study investigates whether reduced optic atrophy 1 (*Opa1*) level promotes apoptosis and retinal vascular lesions associated with diabetic retinopathy (DR). Four groups of mice: wild type (WT) control mice, streptozotocin (STZ)-induced diabetic mice, *Opa1*^+/−^ mice, and diabetic *Opa1*^+/−^ mice were used in this study. 16 weeks after diabetes onset, retinas were assessed for Opa1 and Bax levels by Western blot analysis, and retinal networks were examined for acellular capillaries (AC) and pericyte loss (PL). Apoptotic cells were detected in retinal capillaries using TUNEL assay, and caspase-3 activity was assessed using fluorometric analysis. Opa1 expression was significantly downregulated in retinas of diabetic and *Opa1*^+/−^ mice compared with those of WT mice. Inducing diabetes further decreased Opa1 expression in retinas of *Opa1*^+/−^ mice. Increased cytochrome c release concomitant with increased level of pro-apoptotic Bax and elevated caspase-3 activity were observed in retinas of diabetic and *Opa1*^+/−^ mice; the number of TUNEL-positive cells and AC/PL was also significantly increased. An additional decrease in the Opa1 level in retinas of diabetic *Opa1*^+/−^ mice exacerbated the development of apoptotic cells and AC/PL compared with those of diabetic mice. Diabetes-induced Opa1 downregulation contributes, at least in part, to the development of retinal vascular lesions characteristic of DR.

## 1. Introduction

Loss of retinal vascular cells is a prominent characteristic lesion in early DR [1,2], one of the leading causes of blindness in working-age adults [3]. It has become increasingly evident that mitochondrial abnormalities play a central role in contributing to the pathogenesis of DR [4,5,6,7,8,9,10,11,12,13,14,15,16]. In particular, high glucose (HG)- or diabetes-induced alteration to mitochondrial morphology has been shown to promote apoptotic cell death [4,7,8,10,11,12,13,14,15,16,17]. Specifically, downregulation of a key mitochondrial fusion protein, optic atrophy 1 (*Opa1*), was recently shown to contribute to mitochondrial fragmentation in retinal endothelial cells [8]. However, the extent to which Opa1 downregulation plays a role in promoting apoptotic cell death, leading to the development of retinal vascular lesions associated with DR, is unclear.

Among proteins responsible for regulating mitochondrial dynamics, Opa1 is of particular interest for its role in apoptotic cristae remodeling, mitochondrial fusion, and maintenance of mitochondrial DNA essential for energy metabolism [18,19,20,21,22]. Although mitochondrial fusion is driven by Opa1 in the inner mitochondrial membrane and by mitofusin1/2 (Mfn1/Mfn2) proteins in the outer mitochondrial membrane [23], the effects of Opa1 deficiency were reported to be more severe [23,24,25]. Cellular exposure to apoptotic inducer results in a marked decrease in Opa1 but not Mfn1 or Mfn2 levels [24], and mutations in Opa1 homologs were reported to negatively influence cell growth and survival at a drastically higher rate than mutations in outer membrane fusion proteins, Mfn1 and Mfn2 [23,26]. Control of cytosolic Ca^2+^ levels, NF-kβ signaling, and angiogenic gene expression in endothelial cells are also known to be regulated by Opa1 [27].

The release of cytochrome c is traditionally accepted as a signature of apoptosis. However, studies have shown that cytochrome c release does not always lead to cell death, and that commitment to cell death is regulated downstream of cytochrome c release [28,29,30]. In HeLa cells treated with a caspase inhibitor, cytochrome c release was present despite reduced caspase activity, mitochondrial membrane potential, and decreased cell death [31], suggesting that cytochrome c release may not necessarily translate to apoptotic cell loss. We have shown that a diabetes-induced decrease in retinal Opa1 levels promotes cytochrome c release [8]; however, it has not been documented that this cytochrome c release results in the characteristic development of acellular capillaries and pericyte loss seen in DR. Therefore, in the present study, the effects of Opa1 downregulation and subsequent cytochrome c release in the diabetic retina on the development of retinal vascular cell loss associated with DR were investigated.

To determine whether reduced Opa1 levels promote Bax activation, trigger cytochrome c release, and thereby contribute to the development of retinal vascular cell loss, retinas of *Opa1*^+/−^ mice were examined in this study. Additionally, we evaluated whether inducing diabetes in *Opa1*^+/−^ mice exacerbates the severity of retinal vascular lesions compared with those of diabetic wild-type mice.

## 2. Results

### 2.1. Genotyping of Animals

**Figure 1:**
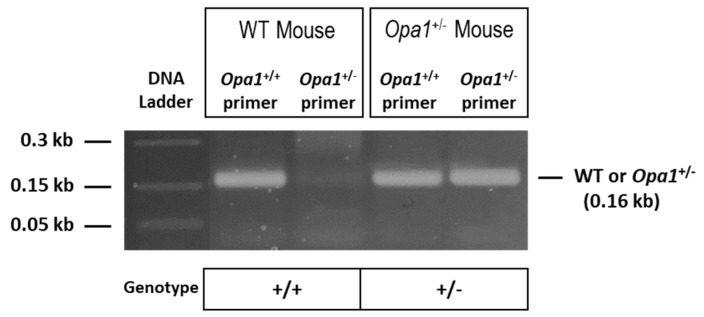
PCR analysis using mice tail tip DNA indicating genotypes of *Opa1* heterozygous knockout (^+/−^) and wild-type (WT) mice. The wild-type *Opa1* allele (*Opa1*^+/+^) and the disrupted allele (*Opa1*^+/−^) are both represented by a band at 0.16kb.

Genotypes of animals used in the present study were confirmed through PCR analysis using DNA derived from the animals’ tail tips. PCR data showed that wild-type (WT) animals (*Opa1*^+/+^) and Opa1 heterozygous knockout (*Opa1*^+/−^) animals exhibit a band at 0.16 kb (Figure 1).

### 2.2. Effect of Diabetes on Opa1 Expression and Distribution in Retinal Capillary Networks

**Figure 2:**
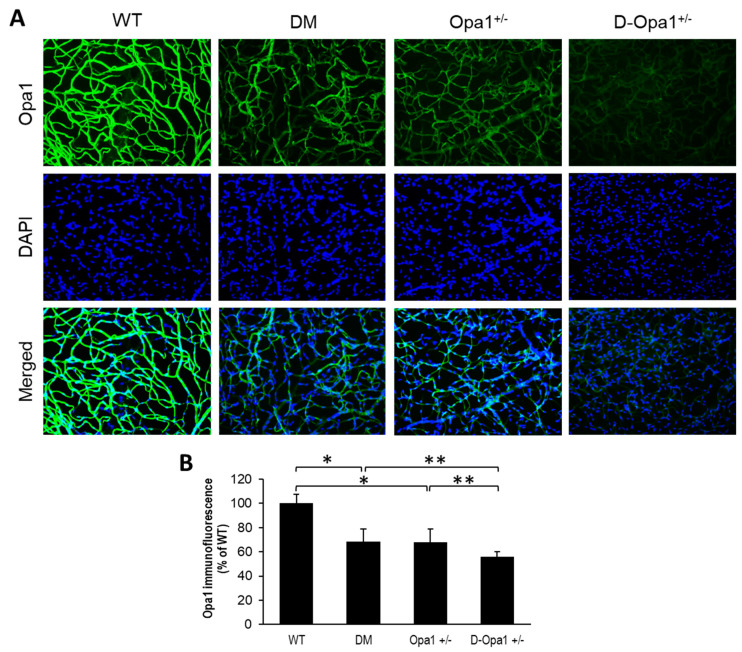
*Opa1*^+/−^ mice exhibit reduced Opa1 immunofluorescence in the retinal capillary network. (**A**) Representative images of Opa1 immunofluorescence (green) and DAPI (blue) in retinal capillaries of wild type (WT) mice and *Opa1*^+/−^ mice; 20x magnification. (**B**) Graphical illustration of cumulative data shows decreased Opa1 immunofluorescence in retinal capillaries of *Opa1*^+/−^ mice compared with that of WT mice. * *p* < 0.01, *n* = 6; ** *p* < 0.05, *n* = 6.

To determine whether the distribution of Opa1 is altered in retinal capillary networks of diabetic animals and Opa1^+/−^ animals, Opa1 immunostaining was performed in retinal trypsin digests (RTDs) from each experimental group. Interestingly, Opa1 immunostaining was significantly decreased in RTDs of diabetic mice compared with that of non-diabetic WT mice (68 ± 10% of WT vs. 100 ± 7% of WT, *p* < 0.01; *n* = 6; Figure 2A,B). Opa1 immunostaining was significantly decreased in RTDs of diabetic *Opa1*^+/−^ mice compared with that of diabetic mice (56 ± 4% of WT vs. 68 ± 10% of WT, *p* < 0.05; *n* = 6; Figure 2A,B) and *Opa1*^+/−^ mice (56 ± 4% of WT vs. 68 ± 11% of WT, *p* < 0.05; *n* = 6; Figure 2A,B).

### 2.3. Diabetes-Induced Opa1 Downregulation Promotes Apoptosis

**Figure 3:**
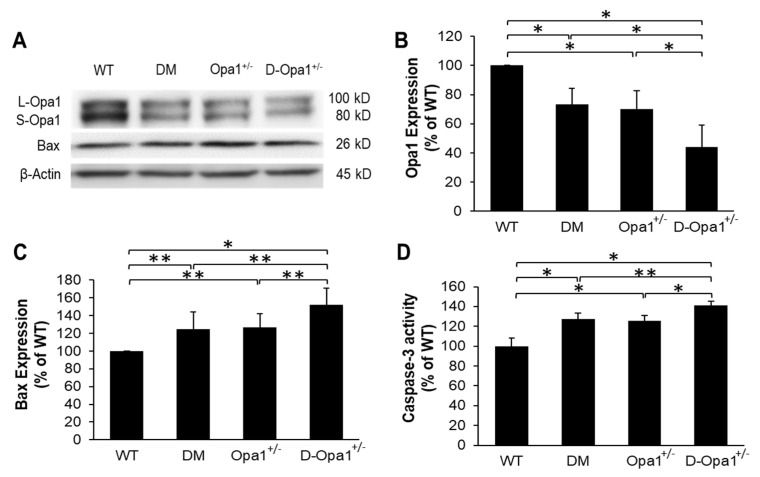
Reduced Opa1 expression increases Bax and caspase-3 activity in *Opa1*^+/−^ mouse retinas. (**A**) Representative WB image shows Opa1 and Bax expression in the retinas of WT, diabetic (DM), *Opa1*^+/−^, and diabetic *Opa1*^+/−^ (D-*Opa1*^+/−^) mice. (**B**) Graphical illustration of cumulative data shows diabetes significantly downregulates Opa1 expression and that D-*Opa1*^+/−^ exhibit further decrease in Opa1 expression. Graphical illustrations of cumulative data suggest reduced Opa1 levels in D-*Opa1*^+/−^ mice promotes a diabetes-induced increase in (**C**) Bax levels and (**D**) caspase-3 activity. Data are expressed as mean ± SD. * *p* < 0.01, *n* = 6; ** *p* < 0.05, *n* = 6.

Western blot analysis showed that Opa1 expression level is significantly downregulated in diabetic mouse retinas compared with that of WT mouse retinas (73 ± 11% of WT, *p* < 0.01; *n* = 6; Figure 3A,B). Additionally, a 1.5-fold decrease in the ratio of short Opa1 isoform (S-Opa1) over long Opa1 isoform (L-Opa1) was observed in diabetic mouse retinas compared with those of non-diabetic WT mouse retinas (Figure 3A). As expected, retinas of *Opa1*^+/−^ mice exhibited a significant decrease in Opa1 expression compared with that of WT mice (70 ± 13% of WT, *p* < 0.01; *n* = 6; Figure 3A,B). In parallel, Opa1 expression was further reduced in retinas of diabetic *Opa1*^+/−^ mice (44 ± 15% of WT, *p* < 0.01; *n* = 6; Figure 3A,B) compared with retinas of diabetic mice. In retinas of diabetic mice, gene expression levels of pro-apoptotic Bax were significantly increased (Bax: 125 ± 20% of WT, *p* < 0.05; *n* = 6; Figure 3A,C) concomitant with increased caspase-3 activity (127 ± 6% of WT, *p* < 0.01; *n* = 6; Figure 3D) compared with those of WT mouse retinas. Interestingly, reduced Opa1 level in retinas of Opa1^+/−^ mice showed a similar increase in Bax expression (126 ± 15% of WT, *p* < 0.05; *n* = 6; Figure 3A,C) and caspase-3 activation (126 ± 6% of WT, *p* < 0.01; *n* = 6; Figure 3D) compared with those of WT mouse retinas. Retinas of diabetic Opa1^+/−^ mice exhibited further increase in Bax expression (152 ± 19% of WT, *p* < 0.05; *n* = 6; Figure 3A,C) and caspase-3 activation (141 ± 4% of WT, *p* < 0.05; *n* = 6; Figure 3D) compared with those of diabetic mouse retinas.

### 2.4. Effects of Reduced Opa1 Levels on Cytochrome c Release

**Figure 4:**
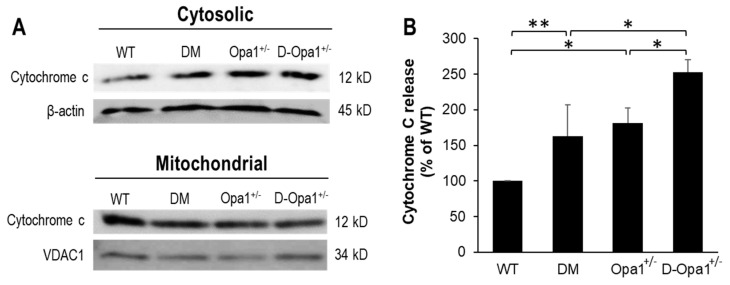
Reduced Opa1 expression in diabetic *Opa1*^+/−^ mouse retinas accelerates cytochrome c release. (**A**) Representative WB image shows cytochrome c levels in cytosolic and mitochondrial fractions in retinas of WT, diabetic (DM), *Opa1*^+/−^, and diabetic *Opa1*^+/−^ (D-*Opa1*^+/−^) mice. (**B**) Graphical illustration of cumulative data shows diabetes and reduced Opa1 level significantly upregulate cytochrome c release, and that further reduction in Opa1 expression in retinas of D-*Opa1*^+/−^ mice results in greater elevation in cytochrome c release. Data are expressed as mean ± SD. * *p* < 0.01, *n* = 6; ** *p* < 0.05, *n* = 6.

Western blot data indicated that retinas of diabetic mice exhibited elevated cytochrome c release compared with that in retinas of non-diabetic WT mice (163 ± 43% of WT, *p* < 0.05; *n* = 6; Figure 4A,B). Additionally, retinas of *Opa1*^+/−^ mice showed elevated cytochrome c release compared with that in retinas of non-diabetic WT mice (181 ± 21% of WT, *p* < 0.01; *n* = 6; Figure 4A,B). Interestingly, further downregulation of Opa1 in retinas of diabetic *Opa1*^+/−^ mice resulted in greater elevation in cytochrome c release (253 ± 18% of WT, *p* < 0.01; *n* = 6; Figure 4A,B) compared with that in retinas of diabetic mice.

### 2.5. Opa1 Downregulation Promotes Apoptotic Death of Vascular Cells in the Diabetic Retina

**Figure 5:**
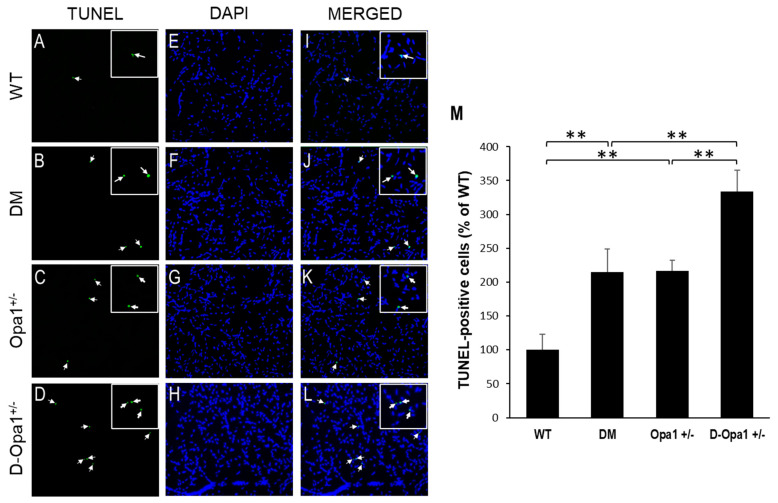
Reduced Opa1 level exacerbates diabetes-induced apoptosis of vascular cells in retinal capillary networks. Representative images show TUNEL-positive cells (arrows) in retinal capillaries of (**A**) WT mice, (**B**) diabetic (DM) mice, (**C**) *Opa1*^+/−^ mice, and (**D**) diabetic *Opa1*^+/−^ mice (D-*Opa1*^+/−^). (**E**–**H**) Corresponding images of DAPI-stained cells in the retinal capillary networks, respectively. (**I**–**L**) Merged images show TUNEL-positive cells are superimposed with DAPI-stained cells; 20× magnification. Insets represent enlarged images of TUNEL-positive cells. (**M**) Graph representing cumulative data shows increased number of TUNEL-positive cells in retinal capillaries of diabetic mice compared with that of WT mice, while retinal capillary networks of D-*Opa1*^+/−^ mice show a further increase in the number of TUNEL-positive cells compared with that of diabetic mice. Data are presented as mean ± SD. ** = *p* < 0.05, *n* = 6.

To assess whether reduced Opa1 expression contributes to apoptotic cell death in retinal vascular cells, TUNEL assay was performed in RTDs from each experimental group. Data indicated that there is a significant increase in the number of TUNEL-positive cells in RTDs of diabetic mice compared with that of non-diabetic WT mice (215 ± 34% of WT vs. 100 ± 23% of WT, *p* < 0.05; *n* = 6; Figure 5A–M). Reduced Opa1 expression alone in RTDs of *Opa1*^+/−^ mice showed a similar increase in the number of TUNEL-positive cells compared with that of non-diabetic WT mice (217 ± 16% of WT vs. 100 ± 23% of WT, *p* < 0.05; *n* = 6; Figure 5A–M). Interestingly, when Opa1 levels were further reduced in diabetic *Opa1*^+/−^ mice, the number of TUNEL-positive cells was significantly increased in RTDs of diabetic *Opa1*^+/−^ mice compared with that of diabetic mice (334 ± 31% of WT vs. 215 ± 34% of WT, *p* < 0.05; *n* = 6; Figure 5A–M).

### 2.6. Diabetes-Induced Opa1 Downregulation Promotes Development of AC and PL

**Figure 6:**
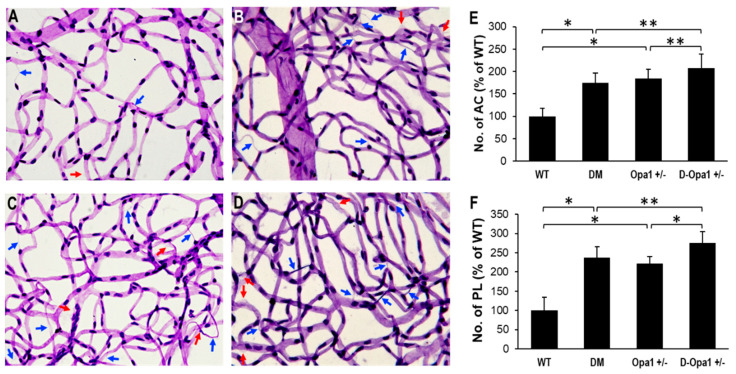
Effects of diabetes or decreased Opa1 level on AC and PL development in mouse retinas. (**A**–**D**) Representative images of retinal capillaries from (**A**) WT, (**B**) DM, (**C**) *Opa1*^+/−^, and (**D**) D-*Opa1*^+/−^ mice show an increased number of AC (blue arrows) and PL (red arrows) in the diabetic mouse retina compared with those of the control mouse retina; 40× magnification. Importantly, the number of AC and PL is increased in retinas of D-*Opa1*^+/−^ mice compared with that of diabetic mice. Graphical illustrations of cumulative data show reduced Opa1 levels in the D-*Opa1*^+/−^ mouse retina exacerbates the development of (**E**) AC and (**F**) PL. Data are expressed as mean ± SD. * = *p* < 0.01, *n* = 12; ** = *p* < 0.05, *n* = 12.

To evaluate the effects of diabetes and reduced Opa1 levels in the development of AC and PL, retinal trypsin digestion was carried out and the numbers of AC and PL between the experimental groups were analyzed. RTD data indicated a significant increase in the numbers of AC and PL in retinal capillary networks of diabetic mice compared with those of non-diabetic WT mice (AC: 175 ± 21% of WT vs. 100 ± 18% of WT, *p* < 0.01; *n* = 12; Figure 6A–F, PL: 237 ± 28% of WT vs. 100 ± 34% of WT, *p* < 0.01; *n* = 12; Figure 6A–F). Reduced Opa1 levels alone in RTDs of *Opa1*^+/−^ mice exhibited a similar increase in the numbers of AC and PL compared with those of non-diabetic WT mice (AC: 184 ± 21% of WT vs. 100 ± 18% of WT, *p* < 0.01; *n* = 12; Figure 5A–F, PL: 221 ± 19% of WT vs. 100 ± 34% of WT, *p* < 0.01; *n* = 12; Figure 6A–F). Interestingly, when Opa1 levels were reduced further in diabetic *Opa1*^+/−^ mice, the numbers of AC and PL were significantly elevated compared with those of diabetic mice (AC: 208 ± 31% of WT vs. 175 ± 21% of WT, *p* < 0.05; *n* = 12; Figure 5A–F, PL: 276 ± 29% of WT vs. 208 ± 31% of WT, *p* < 0.05; *n* = 12; Figure 6A–F).

## 3. Discussion

The present study shows that diabetes-induced Opa1 downregulation stimulates Bax activation, triggers cytochrome c release, and promotes apoptosis in retinal vascular cells, ultimately leading to the development of acellular capillaries and pericyte ghosts associated with DR. It is of interest that reduced Opa1 level alone in the *Opa1*^+/−^ mice was sufficient to yield similar vascular changes seen in retinas of diabetic animals. Moreover, inducing diabetes to the *Opa1*^+/−^ mice accelerated apoptotic cell death, suggesting that severity of DR pathogenesis may be linked to the extent of Opa1 reduction. Additionally, a relative decrease in S-Opa1 over L-Opa1 ratio was observed in diabetic mouse retinas compared with those of non-diabetic mouse retinas. These findings suggest that not only are expression levels of Opa1 reduced, but the processing of mature Opa1 may also be compromised in diabetic retinas.

Recent studies suggest that Opa1 is downregulated in various cell types and tissues under HG or diabetic conditions [32,33,34,35,36]. Cerebral vascular smooth muscle cells grown in HG medium exhibited a significant reduction in Opa1 expression and resulted in increased mitochondrial fragmentation that contributed to compromised contractility and decreased cell proliferation [33]. Similarly, cardiomyocytes grown in HG condition and cardiac tissues from diabetic rats exhibited marked Opa1 downregulation without significant difference in Mfn1 or Mfn2 levels [34]. In a study investigating diabetic neuropathy, Opa1 was found to be significantly downregulated in motor neurons grown in HG condition and in lumbar spinal cord tissues of type 1 diabetic rats [36]. In the type 2 diabetic mouse model, db/db mice, Opa1 levels were significantly reduced in mitochondria of pancreatic islets compared with those of non-diabetic mice [35]. These findings indicate that HG- or diabetes-induced Opa1 downregulation could result in significant negative consequences related to diabetic complications.

Our recent study has shown that HG-induced downregulation of Opa1 increased cytochrome c release and promoted mitochondrial fragmentation in retinal vascular cells of diabetic rodents [8]. However, the consequences of decreased Opa1 level in the diabetic retinas have not been well established. The current study demonstrates that decreased Opa1 level contributes to Bax activation, cytochrome c release, and caspase-3 activation in retinas of diabetic mice as well as in retinas of *Opa1*^+/−^ mice. Recruitment of Bax to the mitochondrial outer membrane is closely associated with mitochondrial fragmentation involving reduced mitochondrial fusion and excess fission [37]. Bax activation, which is sufficient to induce cytochrome c release [38,39], likely acts through mitochondrial outer membrane permeabilization ultimately leading to apoptosis. Other studies have shown Opa1 plays a critical role in maintaining mitochondrial cristae opening [21] and that if these openings are perturbed, cytochrome c localized within the mitochondrial cristae could escape into the cytosol, triggering caspase activation and cell death [24,25]. Findings from our present study indicate that reduced Opa1 level not only compromises mitochondrial inner membrane integrity and leads to cytochrome c release, but also contributes at least in part to mitochondrial outer membrane permeabilization through Bax upregulation, ultimately leading to caspase-3 activation and cell death.

Loss of Opa1 negatively impacts mitochondrial morphology through mitochondrial swelling and development of localized constrictions in the mitochondria leading to mitochondrial fragmentation [40]. Importantly, Opa1 deficiency leads to significant mitochondrial fragmentation, which in turn could impair respiratory function [41]. Mice with decreased Opa1 levels express mitochondrial respiratory deficiency, a selective loss of respiratory complex IV subunits [42] and may ultimately compromise visual function [42]. Opa1 downregulation has also been implicated in contributing to inflammation known to participate in the development and progression of retinal damage in diabetes. Studies have shown that Opa1 deficiency could generate abnormally activated T cells and promote inflammation [43]. More recently, Opa1 ablation was shown to cause muscle inflammation through NF-Kβ activation and enhanced pro-inflammatory gene expression [44]. Taken together, these findings suggest future studies are necessary to address the role of pro-inflammatory effects as well as compromised mitochondrial bioenergetics underlying reduced Opa1 in the context of DR pathogenesis.

Our current study confirms that a decrease in Opa1 levels can promote apoptotic death of retinal vascular cells and lead to the formation of acellular capillaries and pericyte loss characteristic of DR pathogenesis. Moreover, induction of diabetes in *Opa1*^+/−^ mice accelerates apoptotic cell death and capillary degeneration, suggesting that the extent of Opa1 downregulation may be linked to the severity of retinal vascular lesions in DR. Overall findings from the current study suggest that diabetes-induced Opa1 downregulation could be a potential target against the development of retinal vascular lesions in DR.

## 4. Materials and Methods

### 4.1. Animals

The present study was conducted following the guidelines of the ARVO Statement for the Use of Animals in Ophthalmic and Vision Research, and approved by the IACUC Committee of Boston University School of Medicine (PROTO201800411; approved on 9 March 2021). To account for any sex-related differences, both male and female mice were investigated. Specifically, 12 male and 12 female WT C57/BL6J mice obtained from Jackson Laboratory (Bar Harbor, ME), as well as 12 male and 12 female *Opa1*^+/−^ mice with the C57/BL6J strain kindly provided by Dr. Marcela Votruba [42] were utilized to conduct experiments. A detailed methodology on how *Opa1*^+/−^ mice were generated can be found in a study by [42]. Polymerase chain reaction (PCR) using a MasterMix (Promega, Madison, WI, USA) was performed to verify the genotype of the experimental animals used in the study. Primer sequences included in the PCR reaction were as follows: Primer 1, 5′–CTCTTCATGTATCTGTGGTC–3′; Primer 2, 5′–TTACCCGTGGTAGGTGATCATG–3′; and Primer 3, 5′–TTACCCGTGGTAGGTGATCATA–3′. The *Opa1*^+/+^ allele was identified at a band present at 160 bp amplified by primers 1 and 2. The *Opa1*^+/−^ allele was detected at a band present at 160 bp amplified by primers 1 and 3. In the present study, the *Opa1*^−/−^ mice were not included as the genotype is embryonically lethal [42,45].

To induce type 1 diabetes, STZ was injected intraperitoneally at a concentration of 40 mg/kg body weight daily for 5 days in 12 WT mice and 12 *Opa1*^+/−^ mice. Animals representing non-diabetic controls received intraperitoneal injections of citrate buffer as vehicle. To verify the diabetes status in the animals, blood and urine glucose levels were measured 3 days post-STZ injection. Routine blood glucose assessment was performed 3 times per week. Depending on the hyperglycemic status, NPH insulin injections were administered to achieve a level of ~350 mg/dL. After 16 weeks of diabetes onset, animals were sacrificed, blood was collected from each animal and blood glucose and HbA1c levels were measured. Following sacrifice, retinas from each animal were isolated, and total protein extracted from all samples.

### 4.2. Opa1 Expression and Distribution in Retinal Capillary Networks

To study the distribution and expression of Opa1 in the retinal vasculature, retinal capillary networks were subjected to immunostaining with Opa1 antibody. The retinal capillary networks were washed several times with 1× PBS and subjected briefly to ice-cold methanol, followed by additional PBS washes. Then, the RTDs were exposed to a 2% BSA solution diluted in 1× PBS for 15 min at room temperature to block non-specific antibody binding. Following blocking, the RTDs were subjected to a primary antibody solution containing mouse monoclonal Opa1 antibody (1:200 in 2% BSA-PBS solution, Catalog #sc-393296, Santa Cruz Biotechnology, Dallas, TX, USA) and incubated overnight at 4 °C in a moist chamber. The following day, RTDs were washed in PBS and incubated at room temperature with FITC-conjugated rabbit anti-mouse IgG secondary antibody (1:100 in 2% BSA-PBS Solution, Catalog #315-097-003, Jackson ImmunoResearch, West Grove, PA, USA) for 1 h. After three PBS washes, RTDs were counterstained with DAPI, and mounted in SlowFade Diamond Antifade Mountant reagent (SlowFade Diamond; Molecular Probes, Eugene, OR, USA). Digital images were captured, and Opa1 immunostaining was quantified from 10 random representative fields from each RTD, with equal exposure time for all compared panels for each antigen.

### 4.3. Isolation of Cytosolic and Mitochondrial Fractions from Retinas

Retinas of WT mice, diabetic mice, *Opa1*^+/−^ mice, and diabetic *Opa1*^+/−^ mice were isolated, and mitochondrial and cytosolic protein were obtained from the tissues using the Mitochondria Isolation Kit (Thermo Scientific, Waltham, MA, USA) following the manufacturer’s instructions. Briefly, retinal tissues were lysed, centrifuged, supernatant separated and isolated as cytosolic fraction, and centrifuged once more to obtain mitochondrial fractions as previously described [8]. Protein representing mitochondrial and cytosolic fractions were assessed using Western blot analysis for cytochrome c levels.

### 4.4. Western Blot Analysis

Retinal tissues from experimental animals were extracted and protein isolated for analyzing Opa1, Bax, and cytochrome c levels as previously described [8]. The membrane was incubated overnight with mouse monoclonal Opa1 antibody (1:1000, Catalog #sc-393296, Santa Cruz Biotechnology, Dallas, TX, USA), rabbit cytochrome c antibody (1:1000, Catalog#11940, Cell Signaling, Danvers, MA, USA), or rabbit Bax antibody (1:1000, Catalog #2772, Cell Signaling, Danvers, MA, USA) in Tris-buffered solution containing 0.1% Tween-20 (TTBS) and 5% bovine serum albumin. After TTBS washes, the membrane was exposed to a secondary antibody using anti-rabbit IgG, AP-conjugated antibody (1:3000, Catalog #7054, Cell Signaling) or anti-mouse IgG, AP-conjugated antibody (1:3000, Catalog #7056, Cell Signaling) at room temperature and subjected to Immun-Star chemiluminescent substrate (Bio-Rad Laboratories, Hercules, CA, USA). Western blot signals were captured using a digital imager (Fujifilm LAS-4000, Fujifilm, Tokyo, Japan) and densitometry performed using the ImageJ software v1.53i (NIH, Bethesda, MD, USA). B-actin (1:1000, Catalog #4967, Cell Signaling) and VDAC1 (1:1000, Catalog #sc-390996, Santa Cruz Biotechnology, Dallas, TX, USA) antibodies were subsequently used on stripped membranes to verify equal loading of protein in each lane.

### 4.5. Assessment of Caspase-3 Activity

To evaluate caspase-3 activity in retinas of diabetic animals and *Opa1*^+/−^ animals, fluorometric analysis was carried out using a commercially available caspase-3 assay kit (Abcam, Cambridge, UK; Catalog #ab39383). Lysates from retinal tissues were isolated using the kit’s proprietary lysis buffer, incubated on ice for 10 min, and homogenized. Following BCA assay of the retinal tissue lysates, 20 μg of protein from each sample was used to perform fluorometric evaluation of caspase-3 activity. The retinal lysates were mixed with reaction buffer containing DTT (10 mM final concentration) and Acetyl-Asp-Glu-Val-Asp-7-amino-4 trifluoromethylcoumarin (DEVD-AFC) (50 µM final concentration), a fluorogenic substrate specific to caspase-3. The reaction mixture representing each sample was transferred to corresponding wells in a 96-well plate, incubated at 37 °C for 2 h, and subjected to fluorescent excitation and emission at 400 nm and 505 nm, respectively. Specifically, cleavage of the DEVD-AFC substrate is carried out by activated caspase-3 resulting in the formation of free AFC molecules, which can be detected at 400 nm excitation and 505 nm emission [46]. Therefore, relative difference in DEVD-AFC cleavage between experimental groups was used to analyze caspase-3 activity.

### 4.6. Terminal dUTP Nick-End Labeling Assay

To identify cells undergoing apoptosis, terminal deoxynucleotidyl transferase-mediated uridine 5′-triphosphate-biotin nick end labeling (TUNEL) assay was performed using a commercially available kit (ApopTag, Sigma, Temecula, CA, USA) as previously described [47]. Briefly, retinal capillaries were fixed in paraformaldehyde, permeabilized in EtOH-acetic acid, washed in PBS, and incubated in deoxyribonucleotidyl transferase enzyme for one hour at 37 °C. The capillary networks were mounted using antifade reagent (SlowFade Diamond Antifade, Cat#S36963; Invitrogen, Carlsbad, CA, USA) and DAPI. Images from 10 random fields were captured using a digital microscope (Nikon Eclipse; TE2000-S, Nikon, Tokyo, Japan) and analyzed for TUNEL-positive cells.

### 4.7. Retinal Trypsin Digestion and Assessment of Acellular Capillaries and Pericyte Loss

After sacrifice, eyes were enucleated and placed in 10% formalin, and retinas isolated and exposed to 0.5M glycine for 24 h. To isolate retinal capillaries, RTD was performed as described [48]. Briefly, retinas were subjected to 3% trypsin, glia removed through tapping with a single hair brush, and mounted on a silane-coated slide. RTDs were stained with periodic acid-Schiff and hematoxylin as described [49]. Using a digital camera attached to a microscope (Nikon Eclipse; TE2000-S, Nikon, Tokyo, Japan), 10 representative fields were imaged and assessed for AC and PL in a blinded manner. Vessels without endothelial cells and pericytes represented ACs. PL was determined by counting pericyte ghosts, which appear as “empty shells” representing dead pericytes.

### 4.8. Statistical Analysis

Data are shown as mean ± standard deviation. Values representing experimental groups are shown as percentages of the control. The normalized values were subjected to one-way ANOVA followed by Bonferroni’s post hoc test. Statistical significance was considered at *p* < 0.05.

## Data Availability

Data presented in the article are available by request to corresponding author, Sayon Roy (sayon@bu.edu).
